# Severe and Moderate Primary Graft Dysfunction in Adult Heart Recipients

**DOI:** 10.21470/1678-9741-2022-0107

**Published:** 2023

**Authors:** Samuel Padovani Stefen, Davi Freitas Tenório, Guilherme Carvalhal Gnipper Cirillo, Shirlyne Fabianni Gaspar, Karen Amanda Soares de Oliveira, Fábio Antonio Gaiotto, Fabio B. Jatene

**Affiliations:** 1 Instituto do Coração, Hospital das Clínicas, Faculdade de Medicina, Universidade de São Paulo (InCor-HCFMUSP), São Paulo, São Paulo, Brazil; 2 Cardiac Surgery Department, Faculdade de Medicina, Universidade Federal de Goiás (FMUFG), Goiânia, Goiás, Brazil

**Keywords:** Cardiopulmonary Resuscitation, Primary Graft Dysfunction, Heart Transplantation, Tissue Donors, Risk Factors

## Abstract

**Introduction:**

The aims of this study were to determine the incidence of severe and moderate primary graft dysfunction (PGD) in our center, to identify, retrospectively, donors’ and recipients’ risk factors for PGD development, and to evaluate the impact of PGD within 30 days after heart transplantation.

**Methods:**

Donors’ and recipients’ medical records of 64 consecutive adult cardiac transplantations performed between January 2016 and June 2017 were reviewed. The International Society for Heart and Lung Transplantation (ISHLT) criteria were used to diagnose moderate and severe PGD. Associations of risk factors for combined moderate/severe PGD were assessed with appropriate statistical analyses.

**Results:**

Sixty-four patients underwent heart transplantation in this period. Twelve recipients (18.7%) developed severe or moderate PGD. Development of PGD was associated with previous donor cardiopulmonary resuscitation and a history of prior heart surgery in the recipient (*P*=0.01 and *P*=0.02, respectively). The 30-day in hospital mortality was similar in both PGD and non-PGD patients.

**Conclusion:**

The use of the ISHLT criteria for PGD is important to identify potential risk factor. The development of PGD did not affect short-term survival in our study. More studies should be done to better understand the pathophysiology of PGD.

## INTRODUCTION

The outcomes after heart transplantation have improved over the last years, with the mean survival being approximately ten years nowadays^[^1^,^2^]^. Despite this improvement, several factors still contribute to the morbidity and mortality of patients undergoing heart transplantation. In this context, primary graft dysfunction (PGD) is the main cause of early mortality after this procedure^[^3^,^4^]^. The most recent report from the International Society for Heart and Lung Transplantation (ISHLT) Registry reported that 42.6% of deaths within 30 days after heart transplantation were due to PGD^[^1^,^5^]^. Due to the lack of standardized criteria for its diagnosis, the incidence of PGD reported in the literature has varied widely between two and 24%^[^6^,^7^]^. To discuss this issue, the ISHLT recently published a consensus with standardized criteria for PGD^[^8^]^. The ISHLT consensus emphasized that the diagnosis of PGD must be made within 24 hours after completion of the transplantation surgery, and that other discernible causes such as hyper-acute rejection, pulmonary hypertension, or known surgical complications must be ruled out in order to diagnose PGD.

The objectives of this study are to determine the incidence of severe and moderate PGD in our center using the ISHLT criteria, to identify, retrospectively, donors’ and recipients’ risk factors for PGD development, and to evaluate the impact of PGD within 30 days after heart transplantation.

## METHODS

This study was approved by our institutional committee (CAAE: 86764218.1.0000.0068). The medical records of 64 patients undergoing heart transplantation procedures from January 2016 to June 2017 were reviewed. Information about the respective donors was also reviewed. Recipients’ characteristics examined were demographics, etiology of heart failure, and mechanical assistance prior to transplant. Donors’ characteristics included demographics, cause of death, and hemodynamics/use of inotropic support. Procedural characteristics included distance from the donors’ centers to the recipients’ centers, extracorporeal mean time, and cold ischemia time. The ISHLT recently published standardized criteria were used to diagnose PGD (Table 1).

**Table 1:** The International Society for Heart and Lung Transplantation definition scale for primary graft dysfunction (PGD)^[^9^]^.

Left ventricular PGD (PGD-LV)	Mild PGD-LV (one of the following criteria must be met)	LVEF < 40% by echocardiography or hemodynamics with RAP > 15 mmHg, PCWP > 20 mmHg, CI < 2.0 L/min/m^2^ (lasting > 1 h) requiring low-dose inotropes.
	Moderate PGD-LV (must meet one criterion from I and another criterion from II)	I. LVEF < 40% or hemodynamic compromise with RAP > 15 mmHg, PCWP > 20 mmHg, CI < 2.0 L/min/m^2^, hypotension with MAP < 70 mmHg (lasting > 1 h).
		II. High-dose inotropes — inotrope score > 10* or newly placed IABP (regardless of inotropes).
	Severe PGD-LV	Dependence on left or biventricular mechanical support including ECMO, LVAD, BiVAD, or percutaneous LVAD. Excludes requirement for IABP.
Right ventricular PGD (PGD-RV)	Diagnosis requires either both i and ii, or iii alone	i. Hemodynamics with RAP > 15 mmHg, PCWP < 15 mmHg, CI < 2.0 L/min/m^2^.
		ii. TPG < 15 mmHg and/or pulmonary artery systolic pressure < 50 mmHg.
		iii. Need for RVAD.

BiVAD=biventricular assist device; CI=cardiac index; ECMO=extracorporeal membrane oxygenation; IABP=intra-aortic balloon pump; LVAD=left ventricular assist device; LVEF=left ventricular ejection fraction; MAP=mean arterial pressure; PCWP=pulmonary capillary wedge pressure; RAP=right atrial pressure; RVAD=right ventricular assist device; TPG=transpulmonary pressure gradient

* Inotrope score = dopamine (1) + dobutamine (1) + amrinone (1) + milrinone (15) + epinephrine (100) + norepinephrine (100) with each drug dosed in mg/kg/min

Donor heart procurement was performed according to a standard procedure using 3000 ml of Custodiol® solution for preservation of the donor’s heart. All transplant procedures were performed with a bicaval technique, and the same immunosuppression protocol was prescribed to all patients.

## RESULTS

Baseline characteristics of donors and recipients are listed in Tables 2 and 3, respectively. The mean donor age was 33.4 years, and 90.6% of them were male. The etiology of brain death in the majority of cases was head trauma, followed by intracranial hemorrhage; and 78.1% of the donors were in use of inotropic support. There were 24 cases with a > 200 km distance from the donor’s to the recipient’s center. Cold ischemia mean time was 156 minutes (Table 4).

**Table 2:** Donors’ characteristics.

	Overall (64)
Age, years (mean)	33.4
Cause of brain death	
Head trauma	44 (68.7%)
Intracranial hemorrhage	14 (21.8%)
Other	6 (9.5%)
Sex	
Male	58 (90.6%)
Female	6 (9.4%)
Use of inotropic support	50 (78.1%)

**Table 3:** Recipients’ characteristics.

	Overall (64)
Age, years (mean)	49.5
Male sex (%)	62.5
Etiology	
Dilated cardiomyopathy	24 (37.6%)
Ischemic cardiomyopathy	16 (25%)
Chagas disease	20 (31.2%)
Others	4 (6.2%)
LVAD support	
IABP	30 (46.8%)
ECMO	2 (3.3%)
Others	3 (4.6%)
Use of inotropic support	29 (45.3%)

CMO=extracorporeal membrane oxygenation; IABP=intra-aortic balloon pump; LVAD=left ventricular assist device

**Table 4:** Procedural characteristics.

Distance from the donor’s center (> 200 km)	24
Extracorporeal mean time (min)	73
Cold ischemia time (min)	156

Most of the recipients were male (65.5%), with mean age of 49.5 years. The most common etiology of heart failure was dilated cardiomyopathy (37.6%), followed by Chagas disease (31.2%), and ischemic cardiomyopathy (25%). The majority of patients (98.4%) were hospitalized in priority, receiving inotropic support only (45.3%) or some type of left ventricular assist device (LVAD), with intra-aortic balloon pump being the most prevalent (46.8%). Two patients (3.3%) required extracorporeal membrane oxygenation, two patients (3.3%) were in use of LVAD – InCor® support, and only one (1.5%) patient required left Centrimag® support prior to heart transplantation.

A total of 12 (18.7%) patients were diagnosed with moderate or severe PGD using the ISHLT criteria. Of the 12 recipients with PGD, three had right ventricular PGD, six had left ventricular PGD, and three had biventricular PGD. The patients were divided into PGD group and non-PGD group for statistical analysis. The variables compared were recipient-related, donor-related, and procedure-related, and are listed in Table 5.

**Table 5:** Comparison between primary graft dysfunction (PGD) and non-PGD groups.

Variable	Non-PGD	Moderate/severe PGD	*P*-value
Recipients’ variables	Overall (52)	Overall (12)	
Age (mean), years	49.85	45.5	0.44
Gender, female	28 (53.8%)	4 (33.3%)	0.39
Ischemic cardiomyopathy	12 (23.0%)	3 (25%)	0.30
Non-ischemic cardiomyopathy	40 (67%)	9 (75%)	0.35
VAD prior to transplant	28 (54.7%)	6 (50%)	0.75
Prior sternotomy	3 (5.7%)	3 (25%)	0.02
ICU prior to transplant	31 (59.6%)	6 (50%)	0.34
**Donors’ variables**			
Age (mean)	32	30	0.35
Head trauma	34 (65.3%)	7 (58.3%)	0.83
Other cause of death	18 (34.7%)	5 (41.7%)	0.82
Previous CPR	0	2 (16.6%)	0.01
Inotropic support (not > 0.1 ug/kg/min)	46.15 (24%)	7 (58.33%)	0.2
**Procedure-related variables**			
Distance from the donor’s center to the transplant center (> 200 km)	19 (36.5%)	5 (41.5%)	0.4
Ischemic mean time (min)	148	155	0.76

The Chi-square test was used for categorical variables. Comparisons between the groups were made using the paired *t*-test CPR=cardiopulmonary resuscitation; ICU=intensive care unit; VAD=ventricular assist device

With regard to donors’ characteristics, most variables show no significant differences between the PGD group and the non-PGD group. The only possible predictor of PGD was donor’s previous cardiopulmonary resuscitation (CPR) (*P*=0.01). Among recipients’ characteristics, a redo operation was identified as a possible predictor of PGD (*P*=0.02). These findings are shown in Table 5. With regard to postoperative early survival, there were four (6.25%) deaths in the first 30 postoperative days. Among the PGD patients, there was only one (8.3%) death in the early postoperative period, and the other 11 patients (91.6% of all PGD patients) survived the first 30 postoperative days.

## DISCUSSION

Heart transplantation is still the best therapy for patients with advanced heart failure who do not respond to conventional treatments^[^9^]^. Although survival after cardiac transplantation has been improving for the last two decades, the incidence and mortality from PGD is unclear from the literature but it is the most common cause of early mortality^[^8^,^10^]^.

Some previous studies have shown that the incidence of PGD ranges from two to > 20%^[^11^, ^12^, ^13^, ^14^]^. This wide range in the reported incidence of PGD is largely due to inconsistent definitions of PGD across studies. Recently, a report from a consensus conference on PGD proposing standardized criteria for PGD was published^[^8^]^. Based on these standard criteria, the incidence rate of moderate and severe PGD at our institution was 18.7%, which is consistent with other recent studies^[^15^]^. Since we did not report the mild cases, the total incidence of PGD in our center would probably be > 25% of all adult’s transplants. This likely reflects the liberal criteria for PGD proposed by the ISHLT, that recognized that using this definition, a significant number of patients would be diagnosed with PGD^[^16^]^. In our study, we only reported the moderate and severe cases since it is not clear in the literature that mild PGD would somehow impact in morbidity and mortality after heart transplant^[^16^]^.

The pathogenesis of PGD has not been clearly delineated, though its origin is believed to be multifactorial. In one recent study, a longer period of hospitalization and recipients hospitalized at time of transplantation were found to be predictive of PGD^[^17^]^. Our study identified that all PGD recipients were hospitalized at the time of transplantation.

Numerous clinical markers indicating a more severe pre-transplant condition of the recipient, including requirement for inotropic or mechanical support, have been identified as risk factors for PGD suggesting that placing a donor’s heart in a “hostile” recipient environment increases the risk for this complication^[^6^, ^7^, ^8^, ^9^, ^10^, ^11^, ^12^, ^13^, ^14^, ^15^, ^16^, ^17^]^.

The donors’ and recipients’ factors predictive of PGD also vary widely. In the present study, donor’s previous CPR e redo surgery were identified as possible predictive factors for the development of PGD. In others studies, high-dose inotrope use in the donor’s heart was identified as predictive factor^[^15^]^, but we did not found statistical difference regarding this issue.

Although PGD was previously thought to impact survival primarily within a 30-day postoperative period, recent evidence suggests that PGD may affect survival for several months beyond the initial post-transplantation period^[^17^]^. In our study, the in-hospital/30-day mortality for patients with PGD was similar to that for those patients without PGD, that is 8.3% and 5.7%, respectively. A longer follow-up would better evaluate these findings.

### Limitations

This study has several limitations, since it is a retrospective single-center study, with a small number of patients, and with a short 30-day follow-up. More studies should be done applying the ISHLT criteria, mainly in large and contemporary series. This will help to identify potential risk factors of PGD prior to heart transplantation. Also, future prospective studies should be delineated to better understand the pathophysiology of PGD, including possible predictive biomarkers.

## CONCLUSION

The use of the ISHLT criteria for PGD in a large center is important to identify patients at risk for the development of PGD. In our series, PGD did not affect short-term survival. Further studies are necessary to better understand the pathophysiology of PGD.
